# Role of polymorphisms of toll-like receptor (TLR) 4, TLR9, toll-interleukin 1 receptor domain containing adaptor protein (TIRAP) and FCGR2A genes in malaria susceptibility and severity in Burundian children

**DOI:** 10.1186/1475-2875-11-196

**Published:** 2012-06-12

**Authors:** Susanna Esposito, Claudio Giuseppe Molteni, Alberto Zampiero, Elena Baggi, Anna Lavizzari, Margherita Semino, Cristina Daleno, Michela Groppo, Alessia Scala, Leonardo Terranova, Monica Miozzo, Claudio Pelucchi, Nicola Principi

**Affiliations:** 1 Pediatric Clinic 1, Università degli Studi di Milano, Fondazione IRCCS Ca’ Granda Ospedale Maggiore Policlinico, Via Commenda 9, 20122, Milan, Italy; 2 Genetic Unit, Università degli Studi di Milano, Milan, Italy; 3 Department of Epidemiology, Istituto di Ricerche Farmacologiche Mario Negri, Milan, Italy

**Keywords:** Children, cerebral malaria, FCGR2A, malaria, SNPs, toll-like receptors, TIRAP, TLR4, TLR9, uncomplicated malaria.

## Abstract

**Background:**

Malaria caused by *Plasmodium falciparum* is one of the leading causes of human morbidity and mortality from infectious diseases, predominantly in tropical and sub-tropical countries. As genetic variations in the toll-like receptors (TLRs)-signalling pathway have been associated with either susceptibility or resistance to several infectious and inflammatory diseases, the supposition is that single nucleotide polymorphisms (SNPs) of TLR2, TLR4, TLR9, Toll-interleukin 1 receptor domain containing adaptor protein (TIRAP) and FCGR2A could modulate malaria susceptibility and severity.

**Methods:**

This study was planned to make a further contribution to solving the problem of the real role of the most common polymorphisms of TLR4, TLR9, TIRAP and FCGR2A genes in modulating the risk of malaria and disease severity in children from Burundi, Central Africa. All the paediatric patients aged six months to 10 years admitted to the hospital of Kiremba, Burundi, between February 2011 and September 2011, for fever and suspicion of acute malaria were screened for malaria parasitaemia by light microscopy of thick and thin blood smears. In children with malaria and in uninfected controls enrolled during the study period in the same hospital, blood samples were obtained on filter paper and TLR4 Asp299Gly rs4986790, TLR9 G1174A rs352139, T-1486 C rs187084 TLR9 T-1237 C rs5743836, TIRAP Ser180Leu rs8177374 and the FCGR2A His131Arg rs1801274 polymorphisms were studied using an ABI PRISM 7900 HT Fast Real-time instrument.

**Results:**

A total of 602 patients and 337 controls were enrolled. Among the malaria cases, 553 (91.9 %) were considered as suffering from uncomplicated and 49 (8.1 %) from severe malaria. TLR9 T1237C rs5743836CC was associated with an increased risk of developing malaria (p = 0.03), although it was found with the same frequency in uncomplicated and severe malaria cases. No other differences were found in all alleles studied and in genotype frequencies between malaria cases and uninfected controls as well as between uncomplicated and severe malaria cases.

**Conclusions:**

TLR9 T1237C seems to condition susceptibility to malaria in Burundian children but not its severity, whereas none of the assessed SNPs of TLR4, TIRAP and FCGR2A seem to influence susceptibility to malaria and disease severity in this population.

## Background

Malaria caused by *Plasmodium falciparum* is one of the leading causes of human morbidity and mortality from infectious diseases, predominantly in tropical and sub-tropical countries [1]. However, manifestations of the *P. falciparum* infection can vary significantly from patient to patient, ranging from asymptomatic infection to severe life-threatening disease [2]. Together with parasite virulence phenotypes and level of parasitaemia, the host’s immune response to the infectious agent has been considered one of the most important conditioning factors of malaria susceptibility and severity, with the first immune response to *P. falciparum* strictly related to the activity of toll-like receptors (TLRs) [3]. TLRs are a group of trans-membrane proteins, present in monocytes, macrophages and dendritic cells that play a crucial role in the innate immune system. This operates by differentially recognizing pathogen-associated molecular patterns through their extracellular receptor modules and initiating inflammatory signalling pathways through an intracellular domain [4]. Several studies have demonstrated that TLR2, TLR4 and TLR9 are involved in the recognition of *P. falciparum* ligands and that, when encountering the parasite, they elicit a complex cascade of signalling events. These culminate in trans-activation of a repertoire of pro-inflammatory cytokines, such as interferon (IFN)-γ, interleukin (IL)-12 and tumour necrosis factor (TNF) that should favour the elimination of the infectious agent [5-9]. However, it has been reported that excessive serum levels of these pro-inflammatory cytokines can be frequently found in most of the severe malaria cases [10-14]. Consequently, because genetic variations in the TLR- signalling pathway have been associated with either susceptibility or resistance to several infectious and inflammatory diseases, the supposition is that single nucleotide polymorphisms (SNPs) of TLR2, TLR4 and TLR9 could modulate malaria susceptibility and severity [15]. Moreover, because the Toll-interleukin 1 receptor domain containing adaptor protein (TIRAP) mediates downstream signalling of TLR2 and TLR4 inducing pro-inflammatory response and a SNP of this protein has been reported to diminish TLR2 signalling [16], it has been considered possible that genetic variations of TIRAP could modify host response to *P. falciparum* infection. Finally, genetic variants of FCGR2A, an immunoglobulin G receptor, could be associated with an increased risk of malaria because of a significant reduction in its binding capacity for IgG and C reactive protein (CRP) and the consequent reduced phagocytosis of IgG and CRP opsonized structures [17]. However, studies specifically planned to evaluate the importance of genetics in conditioning susceptibility to and clinical manifestations of malaria have reported conflicting results for all these genetic variants [18-31].

This study was planned to make a further contribution to solving the problem of the real role of the most common polymorphisms of TLR4, TLR9, TIRAP and FCGR2A in modulating the risk of malaria and disease severity. It was carried out in Burundi, a Central African country where malaria is highly endemic perennially and for which no data on relationships between genetics and malaria are available at present.

## Methods

### Study population and recruitment

This study, approved by the Ethical Committee of the Fondazione IRCCS Ca’ Granda, Ospedale Maggiore Policlinico, Milan, Italy, and the hospital of Kiremba, Burundi, was carried out in the hospital of Kiremba, a small town located in the district of Ngozi in the northern part of Burundi, between February 2011 and September 2011.

All the paediatric patients aged six months to 10 years admitted to the hospital of Kiremba, Burundi, for fever and suspicion of acute malaria were screened for malaria parasitaemia by light microscopy of thick and thin blood smears, with two independent readers. If informed written or verbal (in case of illiteracy) consent was obtained from a parent or legal guardian, children for whom both readers were positive for the presence of *P. falciparum* were enrolled and grouped according to disease severity in accordance with World Health Organization criteria [32]. In particular, uncomplicated malaria was diagnosed when children had symptoms and signs of malaria (fever, chills, vomiting, headache), *P. falciparum* on blood smear, and no evidence of malaria complications. Severe malaria was diagnosed in presence of cerebral malaria [repeated seizures or coma (Blantyre coma scale ≤2 or Glasgow coma scale ≤8)], circulatory collapse, respiratory distress, or haemoglobin level <5 g/dL. Otherwise healthy children seen in the hospital during the study period for minor surgery with a similar sex and age distribution and without asymptomatic parasitaemia according to the results of the screening with thick and thin films were enrolled as uninfected controls.

Blood samples were obtained from all the children at enrolment, both cases and controls. Blood was collected on filter paper (LTA, Brugherio, MI) for future DNA extraction and testing and kept in a cool, dry place. Samples collected were sent in the central laboratory in the Department of Maternal and Paediatric Sciences of the University of Milan, Italy, by airmail, once a month, for genetic studies.

### DNA extraction and SNP identification

The blood spots on filter paper were cut into little fragments, then incubated for 1 hour in 3- mL lysis buffer at room temperature on an orbital shaker. Paper residues were separated from lysate by brief spinning. DNA from all samples was then extracted with a NucliSens EasyMAG instrument (Biomerieux, Bagno a Ripoli [FI], Italy), using the Specific B protocol. The DNA extracted was quantified using a Nanodrop ND-1000 instrument (Thermo Fisher Scientific, Waltham, MA, USA), with elution buffer as blank. SNP genotyping was then performed on genomic DNA using pre-designed Taqman® SNP Genotyping assays (Applied Biosystems by Life Technologies, Monza, Italy) [33,34]. SNP selection was based on previously reported associations with malaria in populations of different ethnicities. The non-synonymous SNP of TLR4 Asp299Gly (rs4986790, assay code C__11722238_20), the intronic SNP of TLR-9 G(1174)A (rs352139, assay code C___2301953_10), the two promoter SNPs of TLR-9 at positions T(-1486)C (rs187084, assay code C___2301952_10) and T(-1237)C (rs5743836, assay code C__32645383_10), the TIRAP/MAL Ser180Leu (rs8177374, assay code C__25983622_10) and the FCGR2A His131Arg non- synonyimous SNPs (rs1801274, assay code C___9077561_20) were then studied. Reactions and an allelic discrimination analysis were performed on an ABI PRISM 7900 HT Fast Real-time instrument (Applied Biosystems).

### Statistical analysis

Data were analysed using SAS for Windows v. 9.1 (SAS Institute, Cary, NC, USA). Categorical variables are presented as numbers and percentages, and were analysed using contingency tables and the chi-squared or Fisher’s exact test, as appropriate. The Hardy-Weinberg equilibrium (HWE) was performed by comparing the numbers observed of different genotypes with those expected under the HWE for the estimated genotype frequencies and p > 0.05 was in HWE. Genotyping deviations from HWE were assessed under Pearson’s chi-square (*X*^2^) or the likelihood-ratio statistical test, as appropriate [35,36]. Odds ratios and the corresponding 95 % confidence intervals (CI) were calculated to measure the association between genetic variation in selected genes and risk of malaria overall and severe malaria.

## Results

A total of 602 patients (346 males; mean age ± standard deviation, 5.3 ± 4.4 years) and 337 uninfected controls (196 males; mean age ± standard deviation, 5.4 ± 4.3 years) were enrolled. Among uninfected controls, 106 (31.4 %) were admitted for abdominal surgery, 79 (23.4 %) for orthopaedic surgery, 69 (20.5 %) for urogenital surgery, 55 (16.3 %) for plastic surgery and 28 (8.3 % for otolaryngology surgery. Among malaria cases, 553 (91.9 %) were considered as suffering from uncomplicated malaria and 49 (8.1 %) from severe malaria (12, 24.5 %, with cerebral malaria; 37, 75.5 %, with severe anemia). Table 1 shows the allele frequencies for the selected SNPs in *P. falciparum-*infected children and in controls. All SNPs examined were present in the study populations. However, minor allele frequencies for TLR4 Asp299Gly and for TIRAP Ser180Leu were less than 10 % in patients regardless of disease severity (2.1 % to 7.0 % for TLR4 Asp299Gly, 2.1 % to 3.0 % for TIRAP Ser180Leu) and controls (6 % for TLR4 Asp299Gly and 2.4 % for TIRAP Ser180Leu). On the contrary, minor allele frequency for the other SNPs studied exceeded >20 % in all cases, reaching values around 40 % (42.3 %-45.2 %) and 50 % (43.0 %-48 %) for FCGR2A His131Arg and TLR9 G1174A, respectively. TLR9 T1237C rs5743836 C was found significantly more frequently in children with malaria than in uninfected controls (p = 0.04), although no difference was found between children with uncomplicated and those with severe malaria. No other significant difference among Burundian children with malaria and related controls was found as well as between children with uncomplicated and those with severe malaria for all the other allele frequencies studied (P > 0.05 for all the other comparisons).

**Table 1:** Allele frequencies in the study population for the selected SNPs

**Gene and polymorphic alleles**	Allele frequency					
	Uninfected controls (n = 337)	Children with malaria (n = 602)	P value	Children with uncomplicated malaria (n = 553)	Children with severe malaria (n = 49)	P value
**TLR4 Asp299Gly rs4986790**						
Asp	0.94	0.94		0.93	0.98	
Gly	0.06	0.06	0.55	0.07	0.02	0.07
**TLR9 G1174A rs352139**						
G	0.51	0.53		0.52	0.57	
A	0.49	0.47	0.61	0.48	0.43	0.36
**TLR9 T1486C rs187084**						
T	0.75	0.75		0.76	0.72	
C	0.25	0.25	0.94	0.24	0.28	0.45
TLR9 T1237C rs5743836*						
T	0.68	0.63		0.63	0.63	
C	0.32	0.37	0.04	0.36	0.37	0.93
**TIRAP Ser180Leu rs8177374**						
Ser	0.98	0.97		0.97	0.98	
Leu	0.02	0.03	0.50	0.03	0.02	0.59
**FCGR2a H131R rs1801274**						
His	0.45	0.43		0.42	0.45	
Arg	0.55	0.57	0.58	0.58	0.55	0.61

Significant difference between uninfected controls and children with malaria for TLR9 T1237C rs5743836 only. No difference between children with uncomplicated malaria and those with severe malaria.

Table 2 shows the genotype frequencies for SNPs studied comparing children with malaria and uninfected controls, whereas Table 3 summarizes the genotype frequencies for SNPs studied comparing children with uncomplicated malaria and those with severe malaria. For TLR4, TLR9 and FCGR2A frequency of the genetic variants was as expected, whereas genotyping for TIRAP Ser180Leu variants showed slight deviations from what would be expected under the HWE in the control group (p < 0.001). TLR9 T1237C rs5743836CC was associated with an increased risk of developing malaria (p = 0.03), although it was found with the same frequency in uncomplicated and severe malaria cases. No other differences in all the allele and genotype frequencies studied were found between malaria cases and uninfected controls as well as between uncomplicated and severe malaria cases.

**Table 2:** Genotype frequencies comparing uninfected controls and children with malaria for the selected SNPs

Genotype frequency							
**Gene and polymorphic alleles**	Observed		Expected		P (HWE, *X*^2^)	OR (95 % CI)	P value
	Uninfected controls (n = 337)	Children with malaria (n = 602)	Uninfected controls (n = 337)	Children with malaria (n = 602)			
**TLR4 Asp 299Gly rs4986790**							
Asp/Asp	300 (89.0)	528 (87.7)	300 (89.0)	528 (87.7)	Controls = 0.94	1 (reference)	
Asp/Gly	36 (10.7)	72 (11.9)	36 (10.7)	71 (11.8)	Malaria = 0.78	1.14 (0.73-1.79)	0.55
Gly/Gly	1 (0.3)	2 (0.3)	1 (0.3)	3 (0.5)		1.14 (0.06-67.3)	0.92
**TLR9 G1174A rs352139**							
GG	92 (27.3)	166 (27.5)	89 (26.4)	167 (27.8)	Controls = 0.56	1 (reference)	
GA	163 (48.4)	302 (50.2)	169 (50.2)	300 (49.8)	Malaria = 0.88	1.03 (0.74-1.43)	0.87
AA	82 (24.3)	134 (22.3)	79 (23.4)	135 (22.4)	0.91 (0.61-1.34)	0.60	
**TLR9 T1486C rs187084**							
TT	197 (58.4)	342 (56.8)	191 (56.7)	340 (56.5)	Controls = 0.07	1 (reference)	
TC	113 (33.6)	220 (36.6)	125 (37.1)	223 (37.0)	Malaria = 0.57	1.12 (0.83-1.51)	0.43
CC	27 (8.0)	40 (6.6)	21 (6.2)	39 (6.5)		0.85 (0.49-1.49)	0.55
**TLR9 T1237C rs5743836**							
TT	155 (46.0)	245 (40.6)	155 (46.0)	238 (39.5)	Controls = 0.99	1 (reference)	
TC	147 (43.6)	267 (44.4)	147 (43.6)	280 (46.5)	Malaria = 0.22	1.15 (0.86-1.54)	0.34
CC	35 (10.4)	90 (15.0)	35 (10.4)	84 (14.0)		1.63 (1.03-2.60)	0.03
TIRAP S180L rs8177374*							
Ser/Ser	324 (96.1)	567 (94.2)	321 (95.3)	567 (94.2)	Controls = <0.001	1 (reference)	
Ser/Leu	10 (3.0)	35 (5.8)	16 (4.7)	34 (5.6)	Malaria = 0.46	2.00 (0.98-4.09)	0.06
Leu/Leu	3 (0.9)	0	0 (0.0)	1 (0.2)		-	-
**FCGR2a H131R rs1801274**							
His/His	76 (22.6)	108 (17.9)	73 (21.7)	110 (18.3)	Controls = 0.10	1 (reference)	
His/Arg	152 (45.1)	299 (49.7)	159 (47.2)	295 (49.0)	Malaria = 0.72	1.38 (0.96-2.00)	0.07
Arg/Arg	109 (32.3)	195 (32.4)	105 (31.1)	197 (32.7)		1.26 (0.85-1.86)	0.23

Percentages in brackets. CI, confidence interval; HWE, Hardy-Weinberg equilibrium.

**Table 3:** Genotype frequencies comparing uninfected controls and children with malaria for the selected SNPs

	Genotype frequency						
**Gene and polymorphic alleles**	Observed		Expected		P (HWE, *X*^2^)	OR (95 % CI)	P value
	Children with uncomplicated malaria (n = 553)	Children with severe malaria (n = 49)	Children with uncomplicated malaria (n = 553)	Children with severe malaria (n = 49)			
**TLR4 Asp 299Gly rs4986790**							
Asp/Asp	481 (86.9)	47 (95.9)	481 (86.9)	47 (95.9)	Uncomplicated malaria = 0.74	1 (reference)	
Asp/Gly	70 (12.7)	2 (4.1)	69 (12.5)	2 (4.1)	Severe malaria = 0.88	0.29 (0.03-1.16)	0.08
Gly/Gly	2 (0.4)	0 (0.0)	3 (0.6)			-	-
**TLR9 G1174A rs352139**							
GG	149 (27.0)	17 (34.7)	151 (27.3)	16 (32.6)	Uncomplicated malaria = 0.73	1 (reference)	
GA	280 (50.6)	22 (44.9)	276 (49.9)	24 (49.0)	Severe malaria = 0.56	0.69 (0.34-1.43)	0.27
AA	124 (22.4)	10 (20.4)	126 (22.8)	9 (18.4)		0.71 (0.28-1.71)	0.40
**TLR9 T1486C rs187084**							
TT	316 (57.2)	26 (53.1)	315 (57.0)	26 (53.0)	Uncomplicated malaria = 0.60	1 (reference)	
TC	201 (36.3)	19 (38.8)	203 (36.7)	19 (38.8)	Severe malaria = 0.84	1.15 (0.58-2.22)	0.66
CC	36 (6.5)	4 (8.2)	35 (6.3)	4 (8.2)		1.35 (0.32-4.21)	0.59
**TLR9 T1237C rs5743836**							
TT	226 (40.9)	19 (38.8)	219 (39.6)	20 (40.8)	Uncomplicated malaria = 0.16	1 (reference)	
TC	243 (43.9)	24 (49.0)	257 (46.5)	23 (47.0)	Severe malaria = 0.71	1.17 (0.60-2.33)	0.62
CC	84 (15.2)	6 (12.2)	77 (13.9)	6 (12.2)		0.85 (0.27-2.31)	0.74
TIRAP S180L rs8177374*							
Ser/Ser	520 (94.0)	47 (95.9)	520 (94.0)	47 (95.9)	Uncomplicated malaria = 0.47	1 (reference)	
Ser/Leu	33 (6.0)	2 (4.1)	32 (5.8)	2 (4.1)	Severe malaria = 0.88	0.67 (0.07-2.77)	0.59
Leu/Leu	0 (0.0)	0 (0.0)	1 (0.2)	0 (0.0)		-	-
**FCGR2a H131R rs1801274**							
His/His	97 (17.5)	11 (22.5)	99 (17.9)	10 (20.4)	Uncomplicated malaria = 0.62	1 (reference)	
His/Arg	276 (49.9)	23 (46.9)	272 (49.2)	24 (49.0)	Severe malaria = 0.70	0.73 (0.33-1.74)	0.42
Arg/Arg	180 (32.6)	15 (30.6)	182 (32.9)	15 (30.6)		0.73 (0.30-1.85)	0.46

Percentages in brackets. CI, confidence interval; HWE, Hardy-Weinberg equilibrium.

## Discussion

The overall data seem to indicate that none of the SNPs of TLR4, TIRAP and FCGR2A genes studied, assessed in Burundian children, play a role in modulating susceptibility to malaria and disease severity in this population. Regarding SNPs of TLR9, this study suggests that whereas no role may be ascribable to TLR9 G1174A and TLR9 1486 C, TLR9 T1237C seems to be associated with an increased risk of developing malaria, although this SNP is not associated with different disease severity. Despite data regarding association between polymorphisms and severity of the disease have to be evaluated with caution because the total number of severe cases, particular those with cerebral malaria, is too small to permit to draw definitive conclusions, these findings seem to indicate that variation in the characteristics of TLR9 can play a role in conditioning the development of malaria. Consequently, they can be useful in the identification of children at higher risk of developing the disease and those for whom the preventive measures are strongly needed. However, all of these data have to be confirmed with larger studies because in most of the cases data collected with this study are only partially in agreement with those that have been already published at this regard.

Data on TLR4 Asp299Gly are quite different from those reported by other authors who, on the other hand, obtained results largely conflicting with each other. Mockenhaupt *et al.* reported that this polymorphism was associated with an increased risk of severe paediatric malaria without affecting the risk of infection [19]. On the contrary, Basu *et al.* demonstrated that TLR4 Asp299Gly was more common in patients with low parasitaemia and concluded that TLR4 could have a genetic role in controlling the blood infection level in mild malaria and could indirectly suggest a protective effect of TLR4 Asp299Gly against severe disease [20]. Similarly to the data here reported, Zakeri *et al.*[21] and Sam-Agudu *et al.*[18], respectively, reported a similar incidence of this SNP in children with mild malaria and in controls or in patients with cerebral malaria and in those with uncomplicated malaria. However, because the frequency of TLR4 Asp299Gly was always low in this study and in those previously reported, it is difficult to draw firm conclusions on the role of this SNP in susceptibility modulation to malaria and disease severity. On the other hand, different reports have also been published for another TLR4 SNP, Thr399Ile, which some authors have associated with severe malaria, whereas others have observed with similar frequency in malaria patients and in healthy individuals [19-21]. Consequently, further studies are needed to establish whether TLR4 might effectively be involved in *P. falciparum* recognition and host response in humans and whether TLR4 could contribute to the control of infection.

Studies on TLR9 SNPs have also reported conflicting data. Sam-Agudu *et al.*, studying children with cerebral malaria, found that patients with the C allele at -1237 and G allele at 1174 had higher levels of IFN-γ than those without these alleles [18]. Because animal studies have implied a causal role for IFN-γ in the pathogenesis of cerebral malaria and IFN-γ levels were found higher in children with cerebral malaria who died as compared with survivors [12], these authors concluded that these TLR9 SNPs could play a role in modulating malaria severity. On the contrary, Campino *et al.*, despite reporting evidence of cis-variants acting on gene expression, did not find any convincing association between TLR9 SNPs and malaria severity [24]. The data of this study agree only in part with those reported. As found by Ciampino *et al.*[24] but contrary to what was reported by Sam-Agudu *et al.*[18], no association between TLR9 SNPs and malaria severity was found, although a significant association between TLR9 1237 C and disease susceptibility was evidenced. In the past it was suggested that a possible explanation for the different data collected by Ciampino *et al.*[24] and Sam-Agudu *et al.*[18] could be found in the presence of different haemozoin loads in patients studied. TLR9 is located in the endosomal/lysosomal compartment of the cells and recognizes nucleic acids of microbes including that of *P. falciparum*. Haemozoin is a dark-brown haem crystal produced by *P. falciparum* that functions as a carrier for *P. falciparum* DNA and functionally affects TLR9 response to parasite infection activating TLR9 proportionally to its concentration [9]. Several studies have documented that the haemozoin load is higher in children with SM compared with UM [3,37-39]. Unfortunately, these data seem to differ from this hypothesis because TLR9 T1237C was more common in children with malaria than in uninfected controls but had the same frequency in ill children, independent of disease severity. However, because the number of children with TLR9 SNPs was relatively low in all the studies, in this case too further studies are needed.

In this study, contrary to what was reported by some authors [21,25], no protective effect of TIRAP Ser180Leu heterozygosity on susceptibility to and severity of malaria was found. Prevalence of heterozygosity in this study was quite similar to that reported by most of the authors that have studied African individuals. The differences between this study and those by Zakeri *et al.*[21] and Khor *et al.*[25] can be ascribed to differences in number of subjects enrolled. On the other hand, the lack of any relationship between the presence of TIRAP Ser180Leu heterozygosity and protection from malaria was also found by Leoratti *et al.*[23] and by Hamann *et al.*[26]. These latter authors reported a much reduced prevalence of both heterozygosity and homozygosity for this genetic variant in individuals from malaria holo-endemic Ghana and concluded that this finding strongly argued against an expected positive selection of a malaria-protective trait in Africa. However, in all the studies the number of subjects with homozygosity for TIRAP Ser180Leu was too small to be able to draw firm conclusions.

Regarding FCGR2A His131Arg, the data of this study are completely different from those reported by Schuldt *et al.*[27] who found that homozygosity for this SNP was positively associated with severe malarial anaemia but not with cerebral malaria or other major malaria complications. Several other studies have reported contrasting results on the role of this genetic variant on malaria infection and disease [22,28-31]. Different genetic backgrounds could explain these discrepancies. Alleles of FCGR2A and FCGR3B, which are involved in similar biological functions, can be in linkage disequilibrium and, consequently, may interfere with each other’s evolution selection. Moreover, epistatic effects involving variants located in other genes in the FCGR gene cluster on chromosome 1q23-24 are likely to exist. Alternatively, once again it is possible that most of the studies were based on relatively small study groups providing limited statistical power to reveal significant associations with malaria and its complications.

## Conclusions

Knowledge of the relationships between genetics, susceptibility to and severity of infections is essential to identify subjects at higher risk and to develop specific preventive measures. This is particularly import for malaria that remains one of the most important health problem in several geographic areas. This study shows that genetic polymorphisms of some factors involved in the innate immune response to *P. falciparum* infection have no influence in conditioning susceptibility to and severity of malaria in Burundian children. An exception might be represented by TLR9 T127C for which a significant correlation with an increased risk of developing malaria without any role in determining severity was found. This finding, when confirmed with further studies carried out with greater study samples, can permit to identify those subjects for whom preventive measure are strongly needed. Moreover, knowledge that same aspects of the innate immunity can have different efficacy in facing infectious agents can be of value in the preparation of specific vaccines when immune response evoked by the antigens included in the vaccines involves the factors with modified activity. This has to be considered also in the preparation of the developing malaria vaccines.

## Abbreviations

CRP, C reactive protein; HWE, Hardy-Weinberg equilibrium; SNPs, single nucleotide polymorphisms; TIRAP, Toll-interleukin 1 receptor domain containing adaptor protein; TLRs, toll-like receptors.

## Competing interests

The authors declare that they have no competing interest. The study was partly supported by Bando Giovani Ricercatori 2007 (Italian Ministry of Health) and partly by ABM Onlus (that is a non profit organization).

## Authors’ contributions

SE and NP designed the study and co-wrote the manuscript. CGM, AZ, CD, AS and LT carried out the laboratory assays. EB, AL, MS and MG visited patients and controls, collected the samples and entered the data in the database. MM interpreted results of genetic analyses. CP statistically analysed the data. All the authors read and approved the final manuscript.
